# Identification of Proteases and Protease Inhibitors in Allergenic and Non-Allergenic Pollen

**DOI:** 10.3390/ijms18061199

**Published:** 2017-06-05

**Authors:** Barbara Höllbacher, Armin O. Schmitt, Heidi Hofer, Fatima Ferreira, Peter Lackner

**Affiliations:** 1 Deptartment of Molecular Biology, University of Salzburg, A-5020 Salzburg, Austria; hoellbacherba@gmx.at (B.H.); Heidi.Hofer@sbg.ac.at (H.H.); Fatima.Ferreira@sbg.ac.at (F.F.); 2 Faculty of Agricultural Sciences, Georg-August-Universität Göttingen, D-37075 Göttingen, Germany; armin.schmitt@uni-goettingen.de

**Keywords:** pollen transcriptome, protease, protease inhibitor, allergenicity

## Abstract

Pollen is one of the most common causes of allergy worldwide, making the study of their molecular composition crucial for the advancement of allergy research. Despite substantial efforts in this field, it is not yet clear why some plant pollens strongly provoke allergies while others do not. However, proteases and protease inhibitors from allergen sources are known to play an important role in the development of pollen allergies. In this study, we aim to uncover differences in the transcriptional pattern of proteases and protease inhibitors in *Betula verrucosa* and *Pinus sylvestris* pollen as models for high and low allergenic potential, respectively. We applied RNA sequencing to *Betula verrucosa* and *Pinus sylvestris* pollen. After de-novo assembly we derived general functional profiles of the protein coding transcripts. By utilization of domain based functional annotation we identified potential proteases and protease inhibitors and compared their expression in the two types of pollen. Functional profiles are highly similar between *Betula verrucosa* and *Pinus sylvestris* pollen. Both pollen contain proteases and inhibitors from 53 and 7 Pfam families, respectively. Some of the members comprised within those families are implicated in facilitating allergen entry, while others are known allergens themselves. Our work revealed several candidate proteins which, with further investigation, represent exciting new leads in elucidating the process behind allergic sensitization.

## 1. Introduction

In Europe, more than 20% of the population is affected by allergic rhinitis, where more than 50% of the cases are triggered by pollen [1]. The development of an allergic disease is a complex process with many different determinants, from genetic predisposition, over causal allergens to numerous adjuvants [2]. Although several contributing factors have been identified, a comprehensive mechanistic model is still missing. Many of the major allergens in pollen have been identified [3,4,5]. Nonetheless, it is still unclear, why some pollen, e.g., from *Betula verrucosa*, strongly provoke allergies while others, e.g., from *Pinus sylvestris*, do not.

Both species, *Betula verrucosa* and *Pinus sylvestris* are common in the northern parts of Europe and Asia, where *Pinus sylvestris* also extents to some southern areas [6]. Flowering periods start in March for *Betula verrucosa* and in May for *Pinus sylvestris* and can last for several weeks [7]. In term of morphology, pollen of *Betula verrucosa* has a spherical/triangular shape with a diameter of about 25 μm, while *Pinus sylvestris* has saccate pollen grains possessing a pair of air sacs, which leads to a maximum diameter of about 70 μm [8]. Differences in the resulting buoyancy and the related accessibility to a person may have some influence on the allergenicity. Further determinants include the solubility of the pollen derived allergens [9], the presence of microbial contaminants on the pollen surface [10] or chemical modifications of allergens [11] as consequence of air pollution [10].

In the past years, proteases have come to the forefront of allergy research due to their relevance in assisting allergic sensitization [12]. Protease activity first gained attention in the context of allergology upon the discovery that several allergens are proteases in their natural sources. Besides others these are the cysteine proteases Der p 1 from house dust mite [13] and Amb a 11 from ragweed [14] or the serine proteases Der p 3, 6 and 9 from house dust mite [15] and Api m 7 from honeybee [16]. Currently, UniProt/SwissProt alone lists 372 different allergens from 110 different species, where 27 of these allergens are annotated as proteases and 8 are annotated as protease inhibitors.

Inhaled allergens or contact allergens need to pass the epithelial barrier in order to be recognized and processed by dendritic cells. Allergen source derived proteases (ASDPs) were shown to disrupt these barriers. The above mentioned house dust mite allergen Der p 1 was shown to interfere with the tight junctions between epithelial cells and therefore seems to open its own passage to the underlying tissue [17,18]. The disruption of the epithelial barrier was also shown to be performed by ASDPs [19]. Yet other ASDPs have been reported to stimulate mast cells and dendritic cells through protease activated receptors (PARs), with the subsequent release of mediators triggering the innate immune system [20,21,22]. In fact, there are a number of proteases described to be involved in allergenic phenomena. For a review see Matsumura [23].

Protease activity is regulated by distinct protease inhibitors of different specificity. Several authors investigated changes in allergy related processes upon application of protease inhibitors. Suziki and coworkers [24] blocked protease allergens by their inhibitors and observed reduced allergic responses. Inhibition of Der p 1 and Der f 1 with cystatin A was demonstrated to inhibit IL-8 production of keratinocytes [25]. Runswick et al. [26] have shown that proteolytic enzymes derived from pollen, including *Betula verrucosa*, degrade tight junctions. This process could be blocked by inhibitors of serine and cysteine proteases.

These observations point towards a link between allergenic potential and proteases as well as protease inhibitors contained in the allergen sources. We therefore examined the transcriptome from *Betula verrucosa* pollen as a model of high allergic potential and from *Pinus sylvestris* pollen as a model for low allergic potential, for the appearance of proteases and protease inhibitors. Under the assumption that the mRNA levels correlate with the corresponding expressed proteins in the pollen, qualitative or even (semi-)quantitative assessment is feasible.

RNA-Seq has proven to be a useful tool to examine the transcriptome of pollen [27]. A recent study on the highly allergenic common ragweed revealed differentially expressed genes under certain environmental conditions [28]. Here we used RNA-Seq to obtain general gene expression patterns in *Betula verrucosa* and *Pinus sylvestris* pollen, before focusing on proteases and protease inhibitors. In lack of a reference genome, we built a de-novo transcript assembly and subsequently compared the protein functional profiles of the two pollen with each other as well as with the recently published profiles of *Lilium longiflorum* pollen [29]. We then employed a protein family based annotation scheme in order to identify proteases and their inhibitors. A number of highly expressed candidate proteins are further discussed for their potential role in allergenicity.

Finally, we explored possibilities to approach a quantitative exploration of the expressed proteases and inhibitors. This is particularly challenging, as quantification requires mapping to a common reference and is therefore usually only done for samples stemming from the same or closely related organisms. We report two attempts of inter-species comparison using the transcriptome and proteome of *Arabidopsis thaliana*.

## 2. Results and Discussion

**Figure 1:**
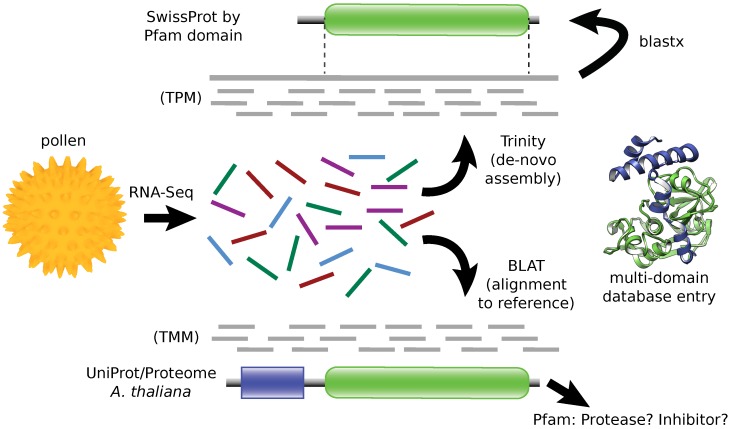
Analysis workflow. Two strategies were implemented: (i) De-novo assembly and mapping the transcript to SwissProt sequences split into Pfam domains. (ii) Direct read mapping to the *Arabidopsis thaliana* reference proteome with subsequent Pfam based assessment. An example of a multi-domain protease is given by the cartoon of Der p 1, which includes a protease domain (green) and a propeptide domain (blue).

Below we first report the outcome of the RNA-Seq procedure and the de-novo assembly using Trinity [30]. We then discuss the generation and comparison of transcriptome functional profiles. Subsequently, we report the Pfam [31] based annotation and identification of proteases and their inhibitors. We proceed with a discussion of our approach of a quantitative comparison. Finally, we report the allergen homolgous transcripts in the two pollen. An overview of the analysis workflow is shown in Figure 1.

### 2.1. RNA Sequencing

RNA-Seq delivered 223 M paired reads for *Betula verrucosa* and 221 M paired reads for *Pinus sylvestris*. We trimmed the reads, removing partially sequenced adapters and low quality bases from the ends in order to ensure that only high quality reads feed into the transcriptome assembly. After quality filtering with Trimmomatic [32] these numbers reduced to 218 M for both pollen. The low loss of read during filtering indicates a good RNA-Seq performance. Basic statistics of the de-novo assembly with Trinity are summarized in Table 1. Abundances were determined using kallisto [33] and are given as estimated counts and TPM (transcripts per million transcripts).

### 2.2. Functional Profiling

Representative transcripts of the Trinity assemblies were annotated through UniProt/SwissProt and then used for functional profiling. In order to get a first indication of whether there are conspicuous differences in the pollen mRNA contents we performed a coarse functional profiling using COG [34] (Clusters of Orthologous Groups) and GO [35] (Gene Ontology) (see Figure S1 in Supplementary File S1 and data in Supplementary Files S2 and S3.) We then compared the distribution of the transcripts to the different COG and GO classes between *Betula verrucosa*, *Pinus sylvestris* and *Lilium longiflorum* pollen [29] as reference. Given the lack of normal distribution of our data, we decided to calculate the non-parametric Spearman rank correlation coefficient to estimate the similarities of the functional profiles (See Table 2. The correlation coefficients between *Betula verrucosa* and *Pinus sylvestris* are 0.98 and higher, regardless of the underlying classifications. There are larger differences between *Lilium longiflorum* on the one hand and *Betula verrucosa*/*Pinus sylvestris* on the other hand. For the 26 COG classes, the correlation *Betula verrucosa*/*Lilium longiflorum* is 0.8 and *Pinus sylvestris*/*Lilium longiflorum* is 0.82. The fraction of transcripts which are related to an active metabolism (energy production and conversion, translation, transcription, signal transduction) are higher in *Lilium longiflorum*. This is intuitively explicable, as in the *Lilium longiflorum* pollen experiment different stages starting from mature pollen to growth of pollen tubes were pooled [29]. With GO biological process, the correlation coefficient between *Lilium longiflorum* and *Betula verrucosa* and *Lilium longiflorum* and *Pinus sylvestris* is 0.52. For GO molecular function and GO cellular location the correlation coefficient is above 0.43 and 0.87.

For all four kinds of classifications, the profiles of *Betula verrucosa* and *Pinus sylvestris* are almost identical (*r* ≥ 0.98). The composition of transcripts is highly similar, showing that there is no a priori bias which would distort our downstream analyses.

### 2.3. Annotation of Proteases and Their Inhibitors

We deployed a protein family based annotation for two reasons. First, the evolutionary distance between *Betula verrucosa* and *Pinus sylvestris* can cause homologues to generate hits to distinct UniProt/SwissProt entries. On the Pfam level, these hits will refer to the same molecular function allowing for a straightforward inter-species comparison. The second reason pertains to the long known problem in functional annotation caused by the existence of protein domains. If a peptidase contains additional, non peptidase domains like adapters or transporters, these domains may give rise to false positive annotations by matches of transcripts to those domains but not to the specific peptidase domain. Possibly less evident is the high content of transposons resembling retro viruses. These encode polyproteins which also contain peptidases. In the context of annotations, a match to those transposons can also lead to false positive hits, if the transcript has similarity to a sequence region outside of the peptidase entity.

We thus decided to employ a domain based strategy to identify peptidases and their inhibitors. Pfam already defines the respective domains and protein families. Selecting the families of interest just by keywords again may lead to numerous false positives. As described in the methods section we used the pfam2go mapping and complemented this by keyword searches. All matching Pfam entries were manually verified. A helpful resource for validation was MEROPS, the Peptidase database [36], which is cross-linked to SwissProt and Pfam. This process resulted in 256 Pfam entries for proteases and 22 for protease inhibitors.

Based on these lists, we extracted all proteases and protease inhibitors from the Pfam *Betula verrucosa*/*Pinus sylvestris* annotations. The proteins were then grouped by Pfam family assignments and ranked by the sum of TPM (transcripts per million transcripts) values. Pfam families where both *Betula verrucosa* and *Pinus sylvestris* had TPM values less than 1 were omitted. The results are summarized in Table 3 and Table 4. Detailed results by SwissProt entry are given in the Supplementary File S4. Below we briefly discuss the most abundant Pfam hits regarding their native functions and possible roles in the context of allergenicity. Note that TPM of *Betula verrucosa* and *Pinus sylvestris* should not be compared in a quantitative manner, as the calculations are based on two separate transriptomes. Ranking by TPM is intended to get a first overview about protein expression of the various protease/inhibitor families.

#### 2.3.1. Proteases

We were able to identify 53 different Pfam protease families (Table 3). The most abundant proteases in both pollen were subtilases (Pfam-ACC: PF00082). Subtilases convergently evolved an Asp/Ser/His catalytic triad, as also found in trypsin serine proteases. They are mostly endopeptidases. Subtilases are over-represented in plants with a large number of different functions, including general protein turnover, developmental processes, responses to environmental signals and cell death [37]. The AllFam [38] database of allergen families lists 23 allergens for this Pfam family, most of them originating from fungi. Ibrahim and coworkers have shown that there is a subtilase in cedar pollen [39], which, when extracted and purified has high IgE-binding capacity and IgE cross-reactivity tested with melon extract. Therefore, the subtilases in *Betula verrucosa* and *Pinus sylvestris* may act as allergen as well.

Second most frequent were matches to the serine carboxypeptidase family PF00450. AllFam lists only two members, Api m 9 from bee and Tri a CPDW-II from wheat. According to AllFam 15% of honeybee sensitized patients have IgE antibodies to Api m 9. In our pollen, in particular carboxypeptidase-like 40 transcript (UniProt-ID: Q0WRX3) was highly expressed.

On 3rd rank we encountered matches to the aspartyl protease family PF14543. This family has eight entries in AllFam originating from fungi, plants and animals.

Next best rank is occupied by the cysteine protease family PF00112. AllFam lists 13 different allergens for this family from plants and animals. To the latter belongs Der p 1 from house dust mite, which is a cysteine protease involved in the disruption of the epithelial barrier [17,18]. Widmer and coworkers [19] have shown that pollen derived proteases are likewise able to degrade tight junctions. Homologues of Der p 1 are also found e.g., in *Arabidopsis thaliana* and *Oryza sativa*. We extracted all Trinity transcripts mapped to PF00112, translated them to protein sequence and created a multiple sequence alignment. The alignment confirmed that the catalytic residues (Cys132, His268 and Asn288 in Der p 1) are conserved in the majority of the mapped transcripts of both *Betula verrucosa* and *Pinus sylvestris*. This shows that the Der p 1 homolog in *Betula verrucosa* and *Pinus sylvestris* is a cysteine protease with potential for epithelial barrier disruption. The TPM values indicate that there is a larger amount accessible in *Betula verrucosa* pollen, which could contribute to higher allergenicity. However, the sheer amount of protease expressed within the pollen is only one of multiple variables determining the levels of accessibility. One has to keep in mind that the amount of pollen a person is exposed to, plays just as important a role. Other mechanistic differences could rely on substrate specificity or different efficiency of the protease activation by auto-cleavage of the activation peptide [40].

Overall, 11 out of the 53 identified Pfams are registered in the AllFam database. Hence, such identified members of these protease families, deriving from the two pollens, have the potential to emulate the properties of allergens.

#### 2.3.2. Protease Inhibitors

In addition to the proteases, we identified members of seven protease inhibitors in the transcriptome. These inhibitors may not only mediate how much a pollen derived protease contributes to the pollen allergenicity, but also interact with host proteases. Of note is, that all of these Pfam families are also listed in the AllFam database. Thus, some of the identified proteins may act as allergen themselves.

Proteases often contain pro-peptides or activation peptides which are cleaved in order to activate the protease. Interestingly, several of these cleaved peptides can act as specific inhibitors [41]. Moreover, several protease inhibitors encoded on separate genes are homologous to these pro-peptides [41].

Most abundant are members of the cysteine-rich secretory protein family (PF00188), which includes the peptidase inhibitor 15 (PI15) and 16 (PI16) proteins. In human, PI15 was shown to have a weak inhibitory effect on trypsin [42]. In plants these proteins are described as pathogenesis-related (PR) proteins with different antifungal, antibacterial and antiviral effects. A functional role as protease inhibitors in plants has to be confirmed.

The inhibitor family I9 (PF05922) contains subtilase inhibitors. The versatile functions of subtilases potentially requires a range of inhibitors which are also present in the pollen.

The families aspartic acid proteinase inhibitor (PF16845) and cystatin (PF00031) belong to the cystatin clan. The aspartic acid proteinase inhibitor is exclusively found in *Betula verrucosa*, cystatin is only expressed in *Pinus sylvestris*. Thus, aspartyl proteases may be inhibited in *Betula verrucosa* and active in *Pinus sylvestris*. In contrast, the higher expressed cysteine proteases in *Betula verrucosa* may be active in *Betula verrucosa* while they are inhibited in *Pinus sylvestris*.

Members of the potato inhibitor I family (PF00280) and serpins (Serine protease inihibitors, PF00079) inhibit trypsin type serine proteases or subtilases [41]. Both are expressed in *Betula verrucosa* and in *Pinus sylvestris*. Finally, the Kunitz STI protease inhibitor (PF00197) was initially identified in legumes, as a potent inhibitor of trypsin. Therefore, it is assumed to protect seeds against herbivores. The two latter protein families were only present at a low abundance.

### 2.4. Towards a Quantitative Comparison

For quantitative analysis two prerequisites need to be fulfilled. First, the equivalent transcripts need to be grouped together. Second, the amount of total RNA measured needs to be comparable. Usually, quantitative studies are performed on samples from tissues or cell types under various experimental conditions but within a certain organism. Then, grouping of equivalent transcripts is relatively easy, especially when a reference genome or trancriptome of the same or a closely related organism is available.

In our case we aim to compare pollen from two organisms, which have an evolutionary distance of 313 M years (according to timetree.org [43]). The nearest, well investigated and most complete genome is the one of *Arabidopsis thaliana*, with a distance of 106 M years from *Betula verrucosa* and 313 M years from *Pinus sylvestris*.

We first used the transcriptome (all types of RNA) from *Arabidopsis thaliana* and applied BLAT [44] to directly assign the quality filtered reads to the transcripts by DNA-DNA matching. The filtering was less stringent than for the de-novo assembly, as sequencing errors are compensated to a large extent by the alignment procedure. Since paired reads are not especially considered in BLAT, we restricted the alignment to the forwards reads only. *Betula verrucosa*, which is more closely related to *Arabidopsis thaliana*, yielded an assignment of 42 M reads to 17,984 different transcripts, while for *Pinus sylvestris* 72 M reads were aligned to 9203 different transcripts, which seems to contradict the evolutionary distances. We proceeded by dissecting matches to protein coding RefSeq mRNAs (NM prefixes) and to non coding RefSeq RNA (NR prefixes, ncRNA) and obtained a quite different picture. For mRNA, *Betula verrucosa* delivers 14 M hits to 17,588 mRNAs, while *Pinus sylvestris* delivers 6 M hits to 8960 mRNAs. These differences are presumably due to higher sequence similarity between *Betula verrucosa* and *Arabidopsis thaliana*. The number of different ncRNAs identified is 396 with 28 M hits in *Betula verrucosa* and 223 with 65 M hits in *Pinus sylvestris*. Among the ncRNA with the highest number of hits are 16S ribosomal RNAs and several uncharacterized RNAs (miscRNAs). It is well known that rRNAs are better conserved than mRNAs. Thus, these data suggest that there is more highly conserved rRNA (and other ncRNA) in *Pinus sylvestris* pollen than in *Betula verrucosa*. As a consequence, the different ncRNA contents in the pollen decrease the TPM values of mRNA to a differing extent between the samples. The relative rank of the mRNA transcripts ordered by TPM is not affected. It needs to be noted that the existence of ncRNA in the final RNA preparations are artefacts. The preparation protocol enriches poly-andenylated RNA, which represents mRNA but also other RNA types. This causes a considerable challenge in comparing the abundances of protein coding transcripts between species via de-novo assemblies.

We then moved to a proteome based strategy, using the Protein-DNA alignment feature of BLAT. This mitigates the problems caused by the different evolutionary distances between our pollen and *Arabidopsis thaliana*, since protein sequences are more conserved than the corresponding nucleotide sequences. The reference was taken from UniProt/Proteomes. As this provides only one representative splice variant for a certain protein, we allow each sequencing read to match only once to a protein. Since we are using a reference proteome, the annotation is an implicit part of the mapping and does not require a second assignment step. The number of reads assigned to a reference RNA or protein respectively, is a measure for its expression level in the sample.

When abundances are calculated in respect to a common reference, the raw counts need to be normalized in order to obtain a comparable metric. We therefore adopted the trimmed mean of M values (TMM) method introduced by Robinson and Oshlack [45]. This method assumes that the expression of most genes is similar and that only a rather small number of genes is differentially expressed. The method finally estimates an effective library size and a weighting factor for each of the two probes to be compared.

In Supplementary File S5 we provide the TMM values for all proteins in the *Arabidopsis thaliana* proteome, which have at least one read assigned in at least one of the two datasets, *Betula verrucosa* or *Pinus sylvestris*. Interestingly, the largest TMM value is assigned to the subtilisin-like protease SBT5.4 (SP-ACC F4JXC5) in *Betula verrucosa*, followed by the subtilisin-like protease SBT5.3 (SPP-ACC Q9ZSP5), thereby highlighting the importance of proteases within the pollen transcriptome. According to TMM the expression of SBT5.4 is 13 times higher than in *Pinus sylvestris*, and the expression of SBT5.3 is 10 times higher than in *Pinus sylvestris*. The two top ranking proteins in *Pinus sylvestris* are the alcohol dehydrogenase class-P protein (SP-ACC P06525, 30 times more than in *Betula verrucosa*) and Glyceraldehyde-3-phosphate dehydrogenase (12 times more than in *Betula verrucosa*).

Using a combination of proteome mapping with BLAT and the concept of TMM could also prove useful for other biological questions which aim to compare protein coding transcripts in the same type of cell or tissue between organisms.

### 2.5. Allergen Homologous Transcripts

The transcriptome data generated in this study provides the basis for many other analyses. In order to complement the study we additional searched for transcripts homologous to known allergens, as the presence of allergens are essential for the development of an allergy. The list of query allergens was taken from UniProt. Out of the 375 query sequences, 198 had a hit in *Betula verrucosa* and 204 in *Pinus sylvestris*, respectively. If we consider only those 298 query allergens which obtain a Blast hit in *Betula verrucosa* or *Pinus sylvestris* and which are also classified in Pfam, we can reduce the number to 52 distinct families. Thus, approximately fifty distinct allergen homologous proteins are contained in the pollen. As expected, the Bet_v_1 family is predominant in *Betula verrucosa*. In *Pinus sylvestris* the glycoside hydrolases family 28 is most abundant. Allergen members of the latter family are Cha o 2, Cry j 2 and Jun a 2. Other abundant transcripts homologous including Cuc m 1 (a serine protease), Hev b 3 (a small rubber particle protein), Fus c 2 (a thioredoxin-like protein) or members of the profilin family are identified, which might have some allergenic potential in general. Detailed results are given in Supplementary File S6.

## 3. Materials and Methods

### 3.1. RNA Sequencing

Total RNA was isolated from the pollen of *Betula verrucosa* (Allergon AB, Thermo Fisher Scientific, Ängelholm, Sweden, batch 012510101) as well as *Pinus sylvestris* (Crystal Laboratory LLC, Luther, OK, USA, batch 209J1J02) as previously described [46] . In brief, 500 mg pollen were homogenized and resuspended in 4.2 M of guanidine thiocyanate, 50 mM of *N*,*N*-Bis(2-hydroxyethyl)-2-aminoethanesulfonic acid pH 7.2, 4 mM EDTA, supplemented with 1% β-mercaptoethanol, 1% of *N*-lauryl-sarkosyl, and 1% *n*-butanol. Following a centrifugation step at 15,000× *g* for 15 min, RNA was extracted from the supernatant using TRIzol LS Reagent (Invitrogen, Carlsbad, CA, USA) according to the manufacturer’s manual.

RNA-sequencing was performed on Illumina’s HiSeq 2500 system (Illumina, San Diego, CA, USA), using the TruSeq RNA LT sample preparation kit (Illumina, San Diego, CA, USA ) and delivered about 220 M paired reads for both *Betula verrucosa* and *Pinus sylvestris*. For a summary see Table 1. Per base sequence quality was encoded in the Phred +33 format and assessed using the tool FastQC [47]. We removed low quality bases from the ends of the reads with Trimmomatic [32] and only continued processing those pairs where both reads passed the checkpoint. As our organisms of interest are not fully sequenced yet, we deployed the Trinity [30] pipeline, which sequentially applies the three tools Inchworm, Chrysalis and Butterfly for de-novo transcriptome assembly. Inchworm decomposes the sequenced reads to form a k-mer library, which is then used to assemble contigs with a greedy algorithm approach. Chrysalis joins contigs with sequence overlap to clusters and creates a de Bruijn graph for each of these clusters. Butterfly then follows this graph and uses the full length reads to resolve ambiguities and construct possible splice forms. Quantification of the assembly was performed with kallisto [33].

Quality parameters and general statistics were obtained by FastQC [47], the fastqutils from NGSUtils [48] and from the RNA-Seq provider’s report.

### 3.2. Annotation

Annotation of the Trinity assembled transcripts: Blastx (*E*-value cutoff 10e ^-3^) was applied to align the transcripts to UniProt/SwissProt (version 11_2016).

### 3.3. Functional Profiling

For functional classification we only considered the longest isoform from each Trinity gene in the *Betula verrucosa* and *Pinus sylvestris* assembly in order to avoid double counting. We call this set “representative Trinity transcripts” below.

GO assignments for molecular function were extracted from the UniProt/SwissProt knowledge base for annotated representative Trinity transcripts. UniProt parsing was performed with Biopython [49]. The GO terms ware traced back up to GO level 2 using the goatools Python library on the GO term hierarchy as defined in the go-basic.obo release 16 January 2017 [35].

For the classification by COG the *Betula verrucosa* and *Pinus sylvestris* representative transcripts were assigned to the COG protein sequences (prot2003-2014.fa) by blastx. Subsequently, the corresponding COG assignments (cog2003-2014.csv) and the COG accession to name mapping (cognames2003-2014.tab) was utilized to count the functional classes. Figure S1 was prepared with ggplot [50].

### 3.4. Identification of Proteases and Protease Inhibitors

For the Pfam (version 30) based annotations we used the uniprot_sprot_pfam30.fa database and the corresponding domain definitions in swisspfam.gz as provided at ftp://ftp.ebi.ac.uk/pub/databases/Pfam/releases/Pfam30.0/ in order to compile a search database consisting only of the Pfam domain sequences. Again, blastx was used to assign the assembled transcripts to these domain sequences.

The collection of peptidase/inhibitor Pfam entries was based on the pfam2go mapping obtained from http://geneontology.org/external2go. This map links GO terms to Pfam entries. We extracted those Pfam-ACCs where a GO ID corresponding to peptidase/inhibitor was assigned. Unfortunately, this mapping is neither complete nor faultless. We first manually confirmed that all indicated Pfam entries received a correct GO assignment using the information and references provided by Pfam. We then performed a keyword search for “(protease or peptidase) and not inhibito” and “(protease or peptidase) and inhibito” respectively in the Pfam-A header as contained in the Pfam-A.full database file. This search was implemented in Python, as Pfam does not support combinatorial queries. Subsequently, all candidates identified by keyword search were manually verified and added to our list if they were indeed a protease/inhibitor.

### 3.5. Quantitative Inter-Species Comparison

For the quantitative comparison the reads were trimmed with Trimmomatic using less stringent parameters than required for the de-novo assembly. These quality filtered reads were then aligned with the BLAT tool to the *Arabidopsis thaliana* reference trancriptome and proteome. The default BLAT parameters have been used. The transcriptome TAIR10, version GCF_000001735.3 was obtained from ftp://ftp.ncbi.nlm.nih.gov/genomes/. This transcriptome contains alternative transcripts for numerous genes. We therefore allowed a read to be aligned to more than one transcript. The proteome was obtained from http://www.uniprot.org/proteomes/ and is built upon genome assembly GCA_000001735.1. The proteome does not contain splice variants and therefore we allowed a read to align only once.

We counted the number of reads mapping to a protein as a measure for its abundance. The TMM calculation was performed in Bioconductor with the edgeR package [51]. The Pfam annotation is based on the protease and inhibitor list described above and on the Pfam UniProt annotation given in the swisspfam.gz file as contained in the Pfam version 30.

### 3.6. Identification of Allergen Homologues

The list of allergens contained in UniProt was obtained from http://www.uniprot.org/docs/allergen. For each allergen, the first appearing isoform (UniProt accession code) was taken as a query for a tblastn search against the Trinity assemblies of *Betula verrucosa* and *Pinus sylvestris*, respectively. The TPM value of the top ranking transcript hit is reported in Supplementary File S6.

## 4. Conclusions

RNA-Seq is a highly valuable tool for the determination of the expression status of a certain biological sample. However, transcript assembly and annotation is challenging. Using domain based annotation avoids false positive hits when searching for distinct functions such as proteases and their inhibitors. However, erroneous classifications in well established databases like Pfam or SwissProt are still observable and need to be resolved manually.

Quantitative comparison of the expression level of certain transcripts between species is problematic when there is no reference genome of a closely related species. This is especially true for the comparison of samples from organisms with increasing evolutionary distance. Using the proteome of an as close as possible model organism and using DNA-Protein alignments can help to achieve this goal. While there are pipelines supporting data analysis within an organism, analysis tools for cross-species RNA-Seq is an orphaned area.

RNA-Seq of *Betula verrucosa* and *Pinus sylvestris* pollen revealed a number of proteases and inhibitors that are candidates for further investigations on protein level. In particular, the Der p 1 homolog should be a promising target.

## Figures and Tables

**Table 1:** RNA-Seq and de-novo assembly statistics.

Attribute	*Betula*	*Pinus*
Total paired reads	223 M	221 M
GC content (%)	47	46
Read length range	35–151	35–151
Read length median	150	150
Paired reads after filtering:	218 M	218 M
Assembled Trinity transcripts:	59,581	85,151
Assembled Trinity genes:	25,263	38,064

**Table 2:** Spearman rank correlation between functional profiles.

	COG		GO-BP		GO-MF		GO-CC	
	Bet	Pin	Bet	Pin	Bet	Pin	Bet	Pin
Lil	0.80	0.82	0.52	0.52	0.43	0.43	0.87	0.87
Bet		0.98		1.00		0.99		0.98

COG: COG Categories, GO-BP: GO Biological Processes, GO-MF: GO Molecular Functions, GO-CC: GO Cellular Components, Bet: *Betula verrucosa*, Pin: *Pinus sylvestris*. Lil: *Lilium longiflorum*.

**Table 3:** List of 53 protease families identified in the *Pinus sylvestris* and *Betula verrucosa* transcriptomes. Sums over transcripts per million transcripts (TPM) of all Trinity transcripts assigned to a certain Pfam family are given.

Pfam ID	Name	∑ TPM Total	∑ TPM *Betula*	∑ TPM *Pinus*
PF00082	Subtilase family	28,858.7	21,782.0	7076.6
PF00450	Serine carboxypeptidase	6259.0	4190.4	2068.6
PF14543	Xylanase inhibitor N-terminal	4466.0	1185.8	3280.2
PF00112	Papain family cysteine protease	4439.9	3076.1	1363.7
PF00227	Proteasome subunit	1451.6	319.9	1131.7
PF14541	Xylanase inhibitor C-terminal	1147.3	880.4	266.9
PF12146	Serine aminopeptidase, S33	871.5	427.1	444.4
PF00443	Ubiquitin carboxyl-terminal hydrolase	699.6	266.3	433.3
PF00026	Eukaryotic aspartyl protease	554.0	240.7	313.3
PF00557	Metallopeptidase family M24	547.5	183.9	363.6
PF01694	Rhomboid family	518.0	328.8	189.2
PF04258	Signal peptide peptidase	469.7	267.2	202.5
PF01434	Peptidase family M41	341.0	199.0	142.0
PF02338	OTU-like cysteine protease	253.4	91.8	161.6
PF05903	PPPDE putative peptidase domain	253.1	106.6	146.6
PF01433	Peptidase family M1	230.4	80.7	149.7
PF00574	Clp protease	228.3	48.9	179.5
PF02127	Aminopeptidase I zinc metalloprotease (M18)	179.6	137.3	42.4
PF00717	Peptidase S24-like	174.8	66.9	107.9
PF01088	Ubiquitin carboxyl-terminal hydrolase, family 1	166.1	35.3	130.8
PF00326	Prolyl oligopeptidase family	146.6	60.9	85.7
PF01965	DJ-1/PfpI family	123.3	13.7	109.6
PF05577	Serine carboxypeptidase S28	106.6	21.7	84.8
PF10502	Signal peptidase, peptidase S26	103.5	66.5	37.0
PF02902	Ulp1 protease family, C-terminal catalytic domain	99.7	20.9	78.8
PF02099	Josephin	99.5	74.6	24.9
PF04389	Peptidase family M28	95.9	11.2	84.8
PF01435	Peptidase family M48	93.2	23.0	70.2
PF13365	Trypsin-like peptidase domain	80.8	39.4	41.3
PF10275	Peptidase C65 Otubain	79.0	14.6	64.5
PF00883	Cytosol aminopeptidase family, catalytic domain	73.5	15.6	57.9
PF00675	Insulinase (Peptidase family M16)	64.7	20.1	44.6
PF00089	Trypsin	60.1	13.7	46.4
PF01470	Pyroglutamyl peptidase	54.5	2.0	52.5
PF01432	Peptidase family M3	52.8	26.0	26.8
PF08325	WLM domain	52.7	39.7	13.0
PF03416	Peptidase family C54	45.6	5.8	39.7
PF09668	Aspartyl protease	39.6	1.8	37.9
PF09768	Peptidase M76 family	36.6	28.8	7.7
PF02163	Peptidase family M50	31.9	4.9	27.0
PF00246	Zinc carboxypeptidase	29.9	20.6	9.4
PF05362	Lon protease (S16) C-terminal proteolytic domain	27.0	2.8	24.2
PF14538	Raptor N-terminal CASPase like domain	26.2	16.9	9.3
PF07910	Peptidase family C78	18.8	3.1	15.7
PF03572	Peptidase family S41	12.2	5.0	7.2
PF03571	Peptidase family M49	11.6	5.3	6.3
PF02586	SOS response associated peptidase (SRAP)	11.6	5.9	5.6
PF00814	Glycoprotease family	10.3	3.7	6.5
PF01457	Leishmanolysin	8.0	0.6	7.4
PF00648	Calpain family cysteine protease	5.5	1.8	3.7
PF00413	Matrixin	5.2	5.2	0.0
PF01551	Peptidase family M23	4.4	0.0	4.4
PF06480	FtsH Extracellular	1.3	0.6	0.6

**Table 4:** Protease inhibitor families. Legend see Table 3.

Pfam ID	Name	∑ TPM Total	∑ TPM *Betula*	∑ TPM *Pinus*
PF00188	Cysteine-rich secretory protein family	19,707.4	19,611.2	96.3
PF05922	Peptidase inhibitor I9	4382.9	2866.2	1516.7
PF16845	Aspartic acid proteinase inhibitor	1328.2	1328.2	0.0
PF00031	Cystatin domain	820.3	0.0	820.3
PF00280	Potato inhibitor I family	154.1	121.2	32.9
PF00079	Serpin (serine protease inhibitor)	84.4	31.7	52.8
PF00197	Trypsin and protease inhibitor	6.5	6.5	0.0

## Supplementary Materials

Supplementary materials can be found at www.mdpi.com/1422-0067/18/6/1199/s1, Supplementary File S1 (pdf) contains Figure S1 and column header description of all Supplementary data Files S2 to S6. Supplementary Files S2 and S3 (tsv) Functional profiles raw data, Supplementary File S4 (tsv) List of proteases and inhibitors including abundances. Supplementary File S5 (tsv) Proteome based annotation including TMM values. Supplementary File S6 (tsv) Transcripts homologous to known allergens. Raw reads are available from NCBI BioProject PRJNA384502.

## Abbreviations

ACC	Accession code
AllFam	Database of allergen families
ASDP	Allergen source derived protease
COG	Clusters of Orthologous Groups
GO	Gene Ontology
ID	Identifier
SP	SwissProt database entry
Pfam	Protein family database entry
TPM	Transcripts per million transcripts
TMM	Trimmed mean of *M* values
