# Supplementary material for: Identification of Proteases and Protease Inhibitors in Allergenic and Non-Allergenic Pollen
Source: Int J Mol Sci. 2017 Jun 5;18(6):1199. doi: 10.3390/ijms18061199 (PMC5486022; doi:10.3390/ijms18061199)
Supplement: Supplementary file 1 [file ijms-18-01199-s001.zip › ijms-196332-SI/Supplementary_file_1.pdf]

Supplementary figure

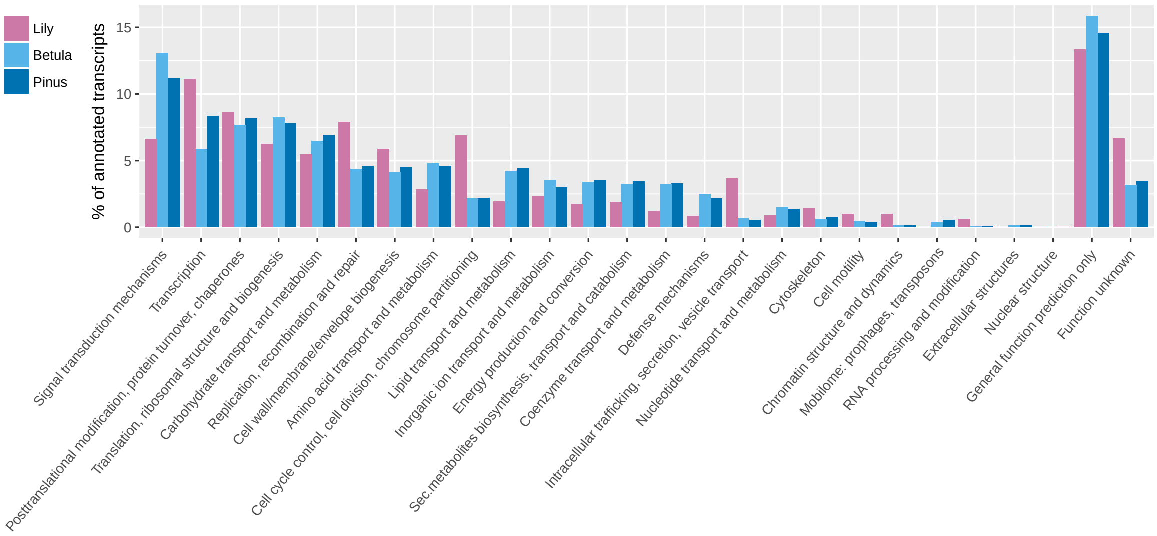

Figure S1: Assignment of *Betula verrucosa*, *Pinus sylvestris* and *Lilium longiflorum* transcripts to COG classes. Frequencies in percent of all assingments are given.

Description of the columns in the supplementary data files

Columns are tab separated.

COG classification (Supplementary File 2):

Field	Meaning
Org	Organism: Betula or Pinus
COG code/name	COG functional class code and name
hits	number of SwissProt matches

GO classification (Supplementary File 3):

Field	Meaning
Org	Organism: Betula or Pinus
GO domain	molecular function, cellular component, biological process
GO term	GO level 1 term
Hits	number of SwissProt matches

Proteases and Inhibitors (Supplementary File 4):

Field	Meaning
Fun	Function: P=Protease, I=Inhibitor
Org	Organism: Betula or Pinus
Pfam Acc	Pfam accession
SP Acc	SwissProt accession
n-hits	number of transcripts matching to this SP Acc
sum(tpm)	corresponding sum of TPM values
sum(estcnt)	corresponding sum of estimated count values

TMM values (Supplementary File 5):

Field	Meaning
UniProt	UniProt accession and ID
TMM Bet	TMM Values for Betula
TMM Pin	TMM Values for Pinus
Prot/Inh	P=Protease, I=Inhibior, PI=both

Allergen homolgous transcripts (Supplementary File 6):

Field	Meaning
Allergen	Allergen name
SP_ACC	SwissProt accession code of the allergen
Pfam	Corresponding Pfam accession code
PI	P=Protease, I=Inhibior, PI=both
TPM_Bet/TPM_Pin	TPM value of the best Blast hit
